# Molecular evidence of piroplasm infection in companion animals in Hunan Province, China

**DOI:** 10.1186/s12917-020-02500-6

**Published:** 2020-08-17

**Authors:** Jinming Wang, Xiaoxing Wang, Hao Sun, Zhaoyun Lv, Youquan Li, Jianxun Luo, Guiquan Guan, Hong Yin

**Affiliations:** 1 State Key Laboratory of Veterinary Etiological Biology, Key Laboratory of Veterinary Parasitology of Gansu Province, Lanzhou Veterinary Research Institute, Chinese Academy of Agricultural Sciences, Xujiaping 1, Lanzhou, Gansu 730046 P. R. China; 2 Jiangsu Co-Innovation Center for the Prevention and Control of Important Animal Infectious Disease and Zoonosis, Yangzhou University, Yangzhou, 225009 P. R. China

**Keywords:** *Babesia*, Pet dog and cat, Feline and canine babesiosis, Nested PCR, China

## Abstract

**Background:**

Feline and canine babesiosis is an important tick-borne disease caused by parasites of the genus *Babesia.* The disease has a worldwide distribution and causes serious health problems in domestic and wild canidae and felidae.

**Results:**

Genomic DNA was isolated from blood samples, which were randomly collected from pet dogs (*n* = 115) and cats (*n* = 25) in Changsha city of Hunan Province, China. Results of nested PCR assay targeting 18S rRNA gene and partial gene sequencing revealed that seven animals were infected with *Babesia* species, five dogs (5/115, 4.3%) and two cats (2/25, 8.0%). Sequence analysis showed that four dogs (3.5%) were positive for *Babesia canis*, and the other one for *Babesia vogeli* (0.87%). The two cats were infected by *Babesia hongkongensis.*

**Conclusions:**

The findings of this study will expand knowledge of the distribution of *Babesia* species and provide important epidemiological information for the control of animal babesiosis in China.

## Background

Feline and canine babesiosis, an important tick-borne hemoprotozoan disease, is prevalent with a worldwide distribution. This disease is caused by several obligate intracellular parasites of the genus *Babesia* and is characterized by fever, anemia, icterus, hemoglobinuria/bilirubinuria, anorexia, weight loss, and weakness caused by destruction of erythrocytes [1, 4, 12, 25]. It has a serious impact on health of domestic and wild felids and canids. So far, at least 13 identified *Babesia* species have been detected in domestic cats or wild felids worldwide, including *Babesia felis*, *Babesia cati*, *Babesia leo*, *Babesia hongkongensis*, *Babesia herpailuri*, *Babesia pantherae*, *Babesia lengau*, *Babesia gibsoni*, *Babesia canis*, *Babesia vogeli*, *Babesia rossi*, *Babesia presentii*, and *Babesia microti*, together with several unidentified *Babesia* species [5, 8, 14].

Most studied clinical cases of feline babesiosis are induced by *Babesia felis*, especially in South Africa. This parasite can infect domestic cats, as well as other felids, such as cheetahs and several wild species [1, 21, 23]. *Babesia cati*, a less pathogenic *Babesia* species, is primarily found in India and results in milder clinical disease [1, 12]. *Babesia leo* was considered to infect lions in South Africa, but it has also been detected in a clinically healthy domestic cat [6, 20]. *Babesia hongkongensis* infection in cats has only been reported in Hong Kong, China [29]. The clinical course of other *Babesia* species in felids has not been well characterized [21].

Compared with feline babesiosis, the pathogens, clinical descriptions and geographic distributions of canine babesiosis are well-documented. Three large *Babesia* species have been identified in dogs, *B. canis*, *B. rossi*, and *B. vogeli*. In addition, an unnamed *Babesia* species that is closely related to *Babesia bigemina*, has been described in North Carolina in the United States [4, 11, 12, 22]. *Babesia vogeli* is widespread in the tropical, subtropical and temperate areas of the world. *Babesia canis* and *B. rossi* are distributed mainly in Europe and Southern Africa, respectively [12]. There are three small *Babesia* species that can infect canids [16]. *Babesia gibsoni* has a worldwide distribution and is a major pathogen of canine babesiosis [32]. *Babesia conradae* has not yet been reported outside California [15]; *Babesia vulpes* has been reported in canids in Spain, Italy, Portugal, Croatia, Germany, and the USA [2, 3, 7].

Since 1985, when Lv et al. diagnosed two cases of canine babesiosis caused by *B. gibsoni* in China [28], this disease has been reported in several provinces across China, including Jiangsu [17], Jiangxi [33], Zhejiang, Anhui, Henan, Shaanxi, Liaoning, Shandong, and Qinghai [24, 26]. These reports have revealed that *B. gibsoni*, *B. vogeli*, and *B. canis* are the causative agents of canine babesiosis in those investigated regions. For feline babesiosis, there is a lack of case reports and pathogen detection in mainland China. However, limited information is available on the prevalence of canine and feline *Babesia* in Hunan Province of China. In the present study, the status of *Babesia* infection was investigated using 115 blood samples from pet dogs and 25 from cats located in Changsha city of Hunan Province, China.

## Results

**Fig. 1:**
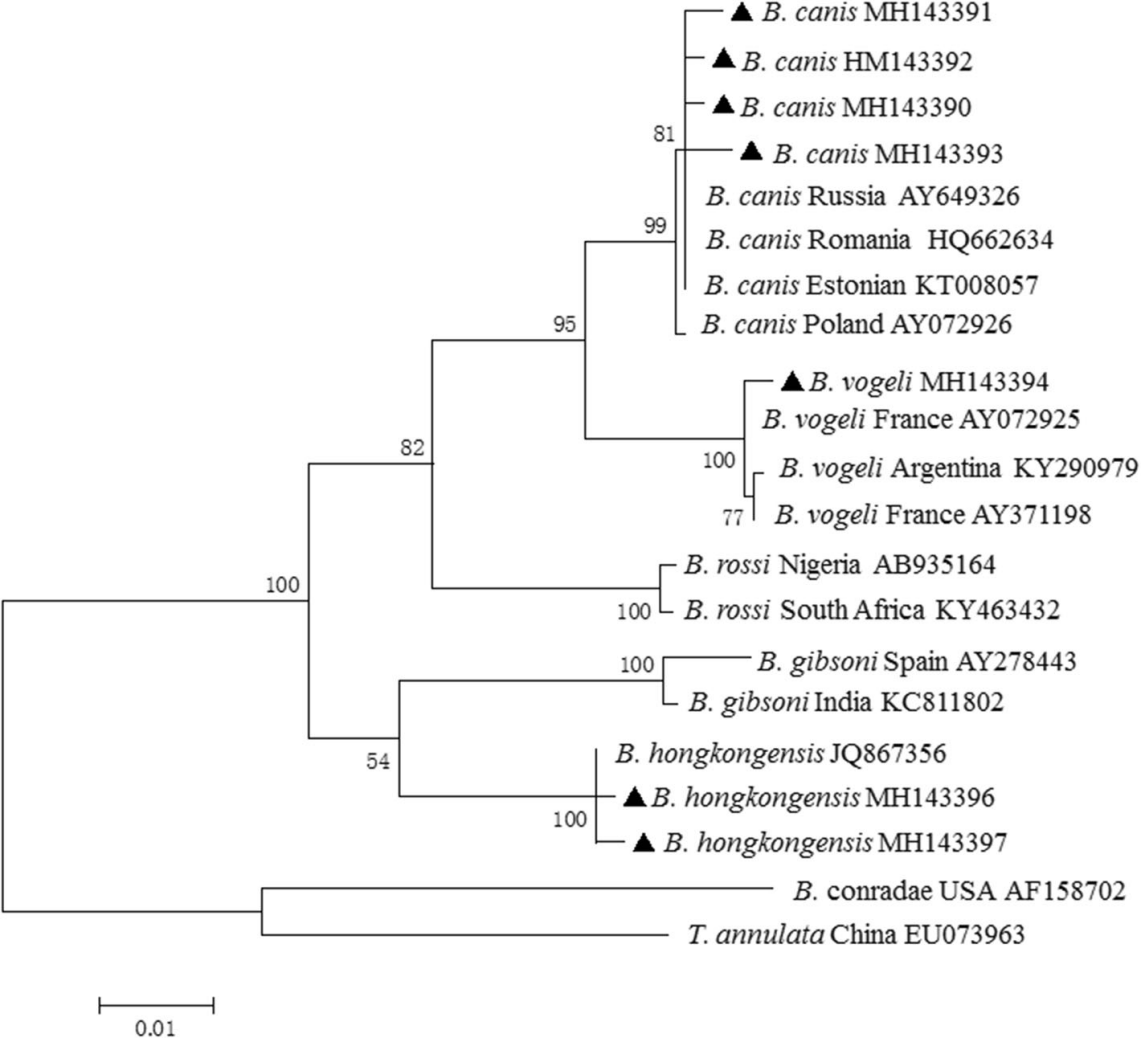
Phylogenetic tree based on the sequences of the *Babesia* 18S rRNA gene (approximately 1400 bp). The tree was constructed using the neighbor joining method of MEGA7 software, and values are given at the nodes. Numbers above the branches demonstrate bootstrap support from 1000 replications. The newly generated sequences in the present study are indicated by bold triangles

Results of the nested PCR revealed that the percentage of blood samples containing piroplasms was 4.2% (5/115) in domestic dogs and 8.0% (2/25) in cats. To classify the *Babesia* species detected in this study, long fragments of the 18S rRNA gene (approximately 1400 bp) were successfully amplified from each positive sample for *Babesia* (Table 1). Results of the sequence analysis were identical to those for the short sequences. The sequences (MH143390–MH143393) shared 99.4–99.9% identity with *B. canis* derived from dogs in Croatia (AY072926) and Estonia (KT008057). Sequences of MH143394 determined in a poodle dog shared 99.6–99.9% identity with *B. vogeli* (KY290979, AY072925, AY371198). The two isolates (MH143396, MH143397) from cats were closely related to *B. hongkongensis* (JQ867356), with 99.6–99.9% sequence identity. Finally, a phylogenetic tree was constructed using the neighbor joining method of MEGA7. The result indicated that the 18S rRNA gene sequences obtained in this study and previously deposited in GenBank were divided into six groups: *B. canis*, *B. vogeli*, *B. rossi*, *B. conradae*, *B. gibsoni* and *B. hongkongensis* (Fig. 1). These data indicate that three *Babesia* spp. infective to dogs or cats were identified in this study.

**Table 1:** Clincial information on the pet animals and the *Babesia* spp. identified in the present study

	Samples ID			Clinical signs	Size of PCR product (bp)	Identified pathogens	Accession number
Species	Animal breed	Age	Sex				
Dog	Miniature schnauzer	11 month	Female	Fever and cough	407, 1405	*B. canis*	MH143375, MH143390
	Japanese Spitz	3 year	Male	lethargy	408, 1419	*B. canis*	MH143376, MH143391
	Chinese Field Dog	5 year	Male	Fever	408, 1406	*B. canis*	MH143377, MH143392
	Border collie	1 year	Female	No signs of babesiosis	407, 1405	*B. canis*	MH143378, MH143393
	Poodle	7 year	Female	Loss of appetite	405, 1404	*B. vogeli*	MH143379, MH143394
Cat	Garfield	3 month	Male	No signs of babesiosis	403, 1413	*B. hongkongensis*	MH143381, MH143396
	British shorthair cat	7 month	Female	No signs of babesiosis	403, 1400	*B. hongkongensis*	MH143382, MH143397

## Discussion

The prevalence of several tick-borne diseases, caused by A*naplasma phagocytophilum, Rickettsia helvetica, Borrelia burgdorferi* and *B. canis*, is closely related to the spatial and temporal distribution of competent vector ticks in specific regions [10, 13]. The majority of investigations of canine piroplasmosis have been performed during the spring or autumn, the seasons of highest vector tick activity. It has been previously reported in China that the prevalence of *B. gibsoni* and *B. vogeli* is 1.5–11.86% and 1.2–11.3%, respectively [9, 18, 30, 33]. In 2019, 130 blood samples were randomly collected from pet dogs in Henan Province to investigate the infection status of piroplasms during the winter. *Babesia canis* infective to dogs was identified for the first time in China, with a prevalence of 8.8% [26].

In this study, to evaluate the infection status of piroplasms, a small scale investigation using a nested PCR assay combined with gene sequencing was performed in companion animals in Hunan Province. The prevalence of *Babesia* spp. infections in dogs and cats were 4.3 and 8.0%, respectively, which indicated that there is a prevalence of canine and feline *Babesia* infection in this area. On the basis of sequence alignment, two canine *Babesia* species (*B. canis*, *B. vogeli*) and one feline *Babesia* species (*B. hongkongensis*) were identified in the dog and cat samples, respectively. No *B. gibsoni* infection was identified in these dogs, but one dog (0.87%) was infected by *B. vogeli.*

The fact that the pet dogs studied have never travelled outside Changsha city suggested that these parasite infections were locally acquired and endemic in this area. A novel *Babesia* species was identified in a free-roaming cat and named *B. hongkongensis* which was previously descried as a feline *Babesia* in China by Wong et al. [29]. In this study, this species was also identified in two cat samples (8.0%). However, the vector tick of *B. hongkongensis* is unknown, and this needs to be investigated in the future.

## Conclusion

This study has demonstrated a prevalence of *Babesia* infection in pet dogs and cats. The three previously recorded canine and feline *Babesia* species (*B. vogeli* and *B. canis* and *B. hongkongensis*) are also prevalent in Hunan Province, China. These data provide valuable information on the distribution of canine and feline *Babesia* species in China.

## Methods

### Sample collection and DNA extraction

Between October 2017 and May 2018, 140 blood samples were randomly collected from pet animals, including 115 dogs (61 males and 54 females, 2 months to 16 years old) and 25 cats (nine males and 14 female, 2 months to 5 years old) in animal hospital located in Changsha in the Hunan Province, China. Blood samples were collected in EDTA-coated vacutainer tubes and transported to the laboratory in iceboxes. Genomic DNA was extracted from 200 μL of each blood sample using a commercial DNA extraction kit according to the manufacturer’s instructions (Qiagen DNA blood mini-kit, Germany).

### Nested PCR for detection of piroplasms infection

A nested PCR (nPCR) that is universal for piroplasms was used to detect piroplasms infective to dogs and cats as previously reported [26, 27, 31]. Briefly, a set of primers (Piro1-S: 5′-CTTGACGGTAGGGTATTGGC-3′, Piro3-AS: 5′-CCTTCCTTTAAGTGATAAG GTTCAC-3′) was applied to amplify large size fragments of 18S rRNA in the first-round PCR [31]. Moreover, genomic DNA of *Babesia bovis* and distilled water were used as positive control and the negative control, respectively. Furthermore, small size fragments were amplified using primers (Piro-A: 5′-TTAAATACGAATGCCCCCAAC-3′ and Piro-B: 5′- ATTACCCAATMCBGACACVGKG-3′) [18, 19, 26, 27, 31]. Positive amplicons were purified using a gel DNA purification kit (Zymo, USA), cloned into the pGEM-T Easy vector (Promega, USA). For each amplicon, three positive clones were sequenced using BigDye Terminator Mix (Genscript, Nanjing, China).

### PCR amplification of *Babesia* 18S rRNA gene fragments

To identify the species of piroplasm infecting pet animals, long fragment*s* of the 18S rRNA gene were amplified from positive samples using a nested PCR assay [26, 27]. For each amplicon, three positive clones were sequenced using the BigDye Terminator Mix (Tsingke Biological Technology, China).

### Sequences analysis

The 18S rRNA gene sequences obtained in this study were subjected to blast analysis on the NCBI website using the BLASTn program. Representative sequences were deposited in the GenBank database.

A phylogenetic tree was inferred by neighbor joining method using MEGA 7.0 software based on the sequences obtained in this study and the 18S rRNA gene sequences of *Babesia* spp. previously submitted to GenBank.

## Data Availability

DNA sequences obtained in this study have been submitted to GenBank database (accession number: MH143375-MH143379, MH143381-MH143382, MH143390-MH143394, MH143396-MH143397).

## Abbreviations

PCR: Polymerase chain reaction assay
rRNA: Ribosomal RNA
EDTA: Ethylene Diamine Tetraacetic Acid
NCBI: National Center for Biotechnology Information
bp: Base pair
